# *HAT*: a high-energy surface X-ray diffraction analysis toolkit

**DOI:** 10.1107/S1600576723000092

**Published:** 2023-02-01

**Authors:** Gary S. Harlow, Sebastian Pfaff, Giuseppe Abbondanza, Zoltan Hegedüs, Ulrich Lienert, Edvin Lundgren

**Affiliations:** a Department of Chemistry and Biochemistry and the Oregon Center for Electrochemistry, University of Oregon, Eugene, OR 97403, USA; b Division of Synchrotron Radiation Research, Lund University, Lund SE-22100, Sweden; c Division of Combustion Physics, Lund University, Lund SE-22100, Sweden; d Deutsches Elektronen-Synchrotron (DESY), Notkestrasse 85, 22607 Hamburg, Germany; Australian Synchrotron, ANSTO, Australia

**Keywords:** data analysis, data reduction, reciprocal space mapping, high-energy surface X-ray diffraction, crystal truncation rods

## Abstract

This work introduces the high-energy surface X-ray diffraction analysis toolkit (*HAT*), an open-source cross-platform software package written in Python to allow the extraction and processing of high-energy surface X-ray diffraction (HESXRD) data sets. Thousands of large-area detector images are collected in a single HESXRD scan, corresponding to billions of pixels and hence reciprocal space positions. *HAT* is an optimized reciprocal space binner that implements a graphical user interface to allow the easy and interactive exploration of HESXRD data sets. Regions of reciprocal space can be selected with movable and resizable masks in multiple views and are projected onto different axes to allow the creation of reciprocal space maps and the extraction of crystal truncation rods. Current and future versions of *HAT* can be downloaded and used free of charge.

## Abstract 2

Computer software for the analysis and extraction of high-energy surface X-ray diffraction data is presented.

## 1. Introduction

**Figure 1:**
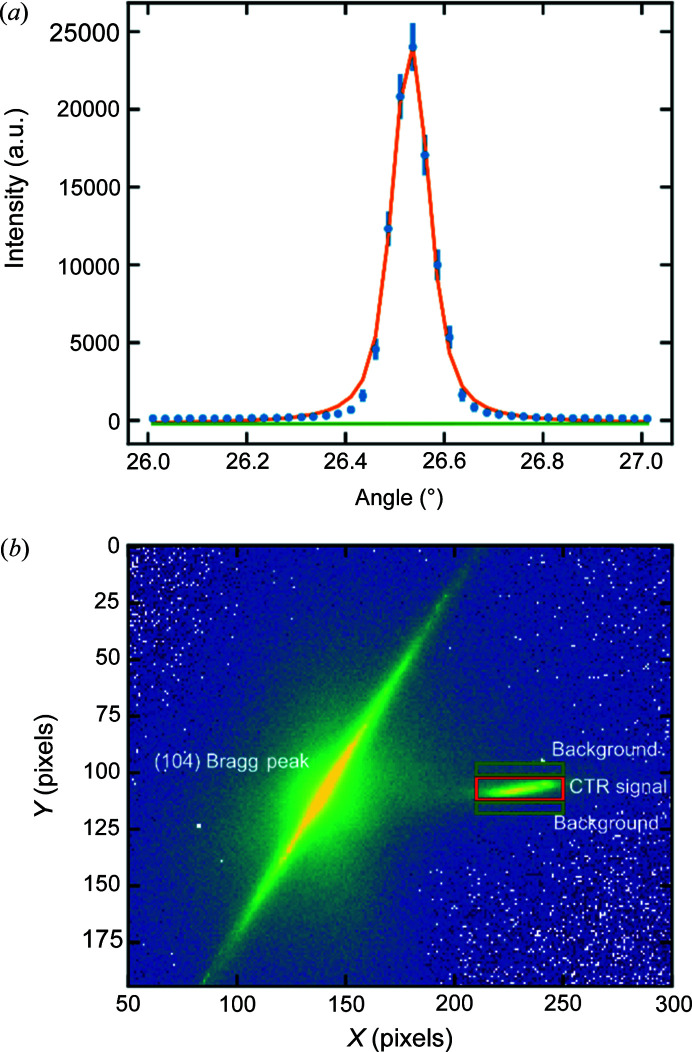
Comparison of how intensity can be extracted via rocking scans or 2D detectors. The sample is a Pt(111) single crystal, measured at *hkl* = (1, 0, 3.8), energy = 14 keV. (*a*) Rocking scan made using the 2D detector as a point detector; data are fitted with a combined Voigt profile (orange) and a linear background (green). (*b*) Small 2D area detector image at *hkl* = (1, 0, 3.8), the CTR signal is indicated by the red box and a background signal can be taken either side (green boxes).

Surface X-ray diffraction (SXRD) is an established technique for the determination of surface structures (Robinson & Tweet, 1992 ▸; Feidenhans’l, 1989 ▸). The surface signal is around six orders of magnitude weaker than the scattering from the bulk structure, and therefore experiments are mostly performed at synchrotron light sources with high brilliance. Historically, point detectors have been used for data acquisition, either by making straight line scans or as a series of ‘rocking scans’ along some direction of interest. These data consisted of a series of small text files, tens of kilobytes in size (experiments totalling hundreds of kilobytes), that can easily be read and stored on most computers. An example of a rocking scan measured through a (1 0) crystal truncation rod (CTR) at *L* = 3.8 for a Pt(111) single crystal is shown in Fig. 1 ▸(*a*); in this case the intensity is plotted as a function of the azimuthal rotation angle. The intensity of the CTR at this position is then simply proportional to the area under the peak and can be corrected by applying a series of correction factors and averaging symmetrically equivalent data points (Vlieg, 1997 ▸). In the popular *ANA-ROD* software package by Elias Vlieg the ‘*ANA*’ part handled these corrections and averaging tasks (Vlieg, 2000 ▸).

Currently, 2D (or area) detectors have almost completely replaced point detectors, as they can simultaneously measure the signal and background, allowing fast scans along a given direction in reciprocal space. Area detectors also make it easier to identify sources of unwanted signals such as powder rings, Bragg peaks and diffuse scattering. These detectors have significantly improved, with newer generations having higher resolution, lower noise, better dynamic range and faster acquisition. Until very recently, most area detectors available at beamlines with sufficient sensitivity for surface diffraction had up to ∼300 000 pixels, with vertical and horizontal pixel sizes between 50 and 150 µm, depending on the type. The data collected from such experiments are then a sequence of images around 0.5–1 MB each (a few gigabytes per experiment). The computational challenge of extracting useful SXRD data from such images is increased considerably compared with that for data measured with a point detector. The most basic solution is to sum a region of interest around the CTR signal and then subtract the sum of some representative background regions; Fig. 1 ▸(*b*) shows an example of such a scheme for the same Pt(111) sample. A more advanced approach could involve fitting some function like a 2D Lorentzian with a sloping background plane. This requires additional competence in image analysis (*e.g.* in Python or *ImageJ*) and is also difficult to automate, often requiring manual inspection of each image. Such a high overhead for simply extracting intensities has significantly increased the ‘measurement to publication’ time of surface diffraction experiments, and in some cases resulted in the data only being partially analyzed.

Fitting profiles or extracting regions of interest can be time consuming but it is also data wasteful, in that the intensity of many of the pixels recorded is simply discarded. A more complete (and fruitful) approach is to perform reciprocal space binning, where the 3D reciprocal space volume is divided into voxels. The intensity of each pixel, in each image after assignment of reciprocal space coordinates, is placed in the corresponding voxel or ‘bin’; this is essentially a 3D histograming process. *BINoculars *from the ESRF is a very successful example of such an approach which has been implemented across several beamlines (Roobol *et al.*, 2015 ▸). The software allows extraction of CTRs, projection of data onto given planes and other measurements such as powder diffraction to be extracted; it has also recently been used for analyzing high-energy surface X-ray diffraction (HESXRD) data (Fuchs *et al.*, 2020 ▸). However, it has proved somewhat difficult for users to implement *BINoculars* themselves, away from the synchrotron. One reason for this is that the software requires a ‘backend’ script for each individual beamline setup and experimental geometry, and the documentation for this is limited to several lines of comments in a Python file. It is also very resource inefficient; it uses a dispatcher model that allows calculations to be distributed over multiple cores or a cluster, but then most of those calculations occur in native Python which is very slow for the types of calculations performed. Another problem with *BINoculars* is that development has stopped, with the last update implemented over 3 years ago.

Gustafson *et al.* (2014 ▸) demonstrated that when using a larger-area detector (∼2000 × 2000 pixels) and higher X-ray energies (60–100 keV) large regions of reciprocal space can be measured with surface sensitivity (if the Bragg peaks are masked by placing beam stops over them on the detector). Although this technique is, in principle, no different from standard SXRD it does offer several advantages and disadvantages, some of which we list in Table 1 ▸. We have also published a few reviews and discussions on the technique (Hejral *et al.*, 2020 ▸; Shipilin *et al.*, 2014 ▸; Harlow *et al.*, 2020 ▸). However, the use of large-area detectors, with many more pixels, further increases the amount of data collected; each image is now up to 40 MB (depending on whether 16 or 32 bit integers are used), and a single sample rotation can be ∼30 GB of data (total experimental data during beam time can be in the terabytes).

The purpose of this paper is to introduce the HESXRD toolkit *HAT*, a piece of software that makes the extraction of useful data from HESXRD data sets relatively straightforward and fast. A graphical user interface (GUI) enables exploration of the data sets on a moderately fast laptop or desktop PC (we recommend at least 64 GB of RAM), allowing various reciprocal space slices and profiles to be extracted using draggable masks. Reciprocal space binning calculations like those carried out in *BINoculars* can be performed and are accelerated using the *Numba* library (Lam *et al.*, 2015 ▸), which can run on either the CPU or a Compute Unified Device Architecture (CUDA)-capable GPU.

## 2. Implementation

**Figure 2:**
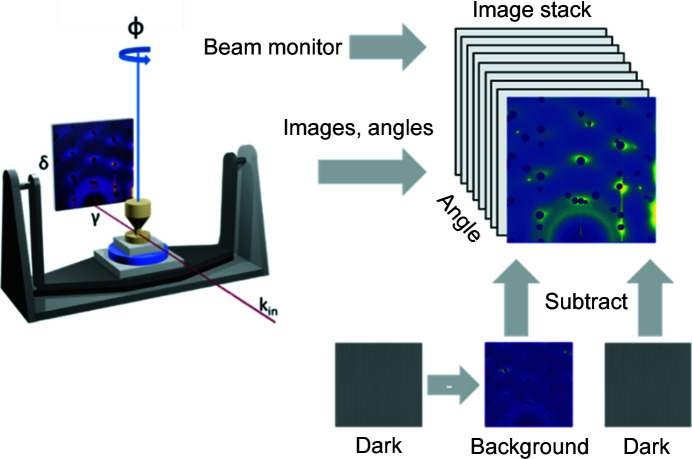
Schematic representation of a typical data set from an HESXRD experiment. A stack of images is collected in the experiment; this can be operated through subtraction of dark images and backgrounds as well as rotation and flipping. Each image corresponds to a different sample angle.

Conducting an HESXRD experiment is, in principle, straightforward: one aligns a single crystal on a diffractometer so that the surface normal is parallel to the phi axis of rotation (*i.e.* perpendicular to the X-ray beam) and then a grazing-incidence angle (typically just above the critical angle) is chosen. Large-area detector images can then be collected while the sample is rotated about the surface normal. Since the Bragg peaks of the single crystal are many orders of magnitude brighter than the CTRs of interest, beam stops such as tungsten pieces attached to magnets are used to block out the intense Bragg peaks. We refer to each image collected during the rotation of the sample as a ‘frame’, and this corresponds to a particular sample rotation angle (ϕ) recorded by the diffractometer encoder. The whole set of images is called an ‘image stack’. Depending on the detector type, we may wish to subtract a dark image from each frame. Furthermore, a background image, such as the sample environment without the sample, can be subtracted and this may also have its own dark image. Each image collected can also be normalized to a beam intensity monitor, such as an ion chamber or the synchrotron ring current. This image stack representation is presented schematically in Fig. 2 ▸. *HAT* implements the image stack as a Python class to allow the greatest degree of flexibility. For instance, it is possible to construct a composite detector image consisting of two detectors side by side with a small gap between them. This makes no difference to the rest of the *HAT* software (in the future we hope to extend this to support more combinations). The image stack class supports initial binning of the frames to a smaller number of pixels (this is useful if the computer has insufficient memory), transformations such as flipping/rotation and intensity offsets/scaling. *HAT* makes extensive use of the *PyQtGraph* library to provide many features (Campagnola, 2022 ▸), *e.g.* the *Numba* library for acceleration (Lam *et al.*, 2015 ▸).

### 2.1. Detector view

**Figure 3:**
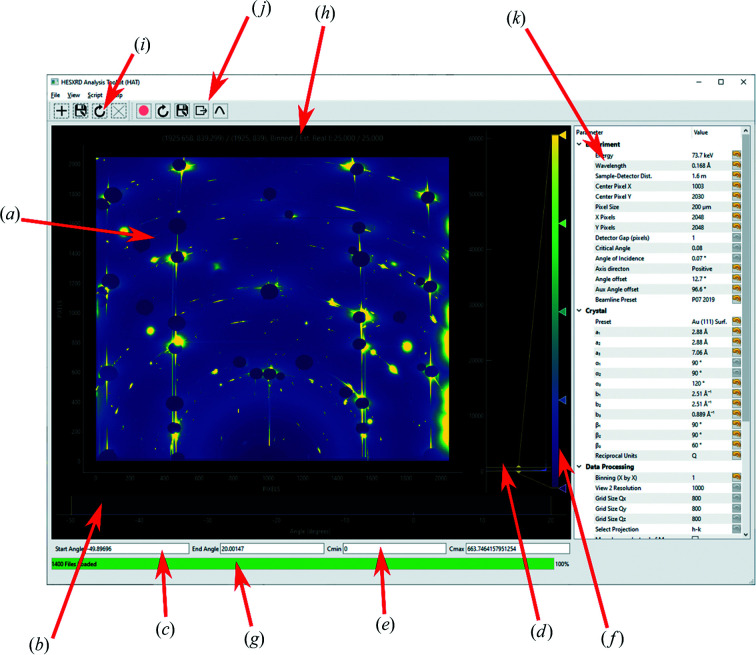
Overview of the *HAT* GUI. (*a*) Image stack view, either maximum intensity for each pixel or summed intensity; (*b*) angle range slider; (*c*) start/end angle; (*d*) color slider; (*e*) color min/max; (*f*) color map picker; (*g*) progress bar and user messages; (*h*) hover coordinates and intensity; (*i*) mask tools; (*j*) region of interest tools; (*k*) parameter tree.

**Figure 4:**
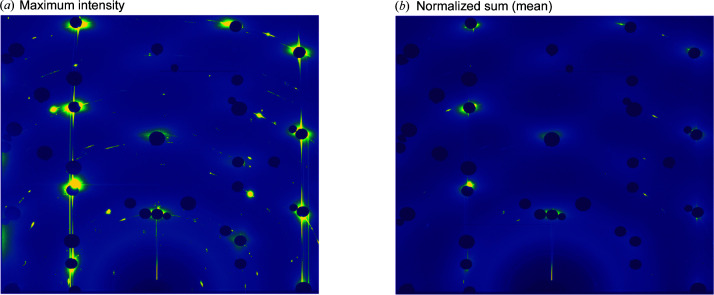
Comparison of maximum intensity versus mean intensity. (*a*) Maximum pixel intensity image and (*b*) normalized summed (mean) intensity image across a 26° rotation for an Au(111) electrode in 0.1 *M* NaOH at 0.1 V (versus RHE). Both images are plotted on the same color scale. The semi-circles are powder rings, and the dark circles are where beam stops have been placed to protect the detector from high-intensity Bragg reflections from the bulk Au(111) single crystal. Running through the masked Bragg reflections are the CTRs; these arise from the termination of the Au(111) crystal. To the side are super-structure rods from the surface reconstruction (closest rods to the edge of the detector). These arise only from the top layer of atoms which have a different periodicity.

Fig. 3 ▸ shows a screenshot of the current version of the *HAT* GUI. The area labeled  ▸(*a*) is the viewport from which the user can choose between several different views using the ‘View’ menu. The view shown in Fig. 3 ▸ is the ‘Detector View’ and simply shows either the average or maximum values across the selected angular range of the image stack. The reason one might prefer the maximum intensity over the average intensity is that, if one sums the intensity, the background and noise are also included in this sum, making it difficult to separate the signal of interest which is only present in a small number of frames. This is illustrated in Fig. 4 ▸ for a 26° rotation from an Au(111) surface in an electrochemical cell containing 0.1 *M* NaOH at a potential of 0.1 V (versus RHE); the full 72° rotation collected on this sample will later be used as an example throughout the manuscript. In the summed image [Fig. 4 ▸(*b*)] the background dominates, whereas the CTRs are clearly visible as straight vertical lines between the beam stops in the maximum image [Fig. 4 ▸(*a*)]. Next to the CTRs are straight lines due to herringbone surface reconstruction. The area selected is chosen either by moving the sliders labeled (*b*) in Fig. 3 ▸ or by changing the ‘Start Angle’ and ‘End Angle’ values (*c*). Similarly, the color scale can be adjusted either with the sliders (*d*) or by changing the ‘*C*_min_’ and ‘*C*_max_’ values (*e*). The color mapping can be chosen by right-clicking on the widget (*f*). User messages and the progress of certain operations are given in the status bar (*g*). The coordinates plus intensities of individual pixels (when hovered over) are displayed at the top (*h*). Various tools such as box profiles and mask tools are accessible from the tool bars (*i*) and (*j*), and experimental parameters can be set in the parameter tree (*k*). Box profiles (a line profile summed along a second axis) can be extracted in any view to show how the intensity varies along any given direction. These can also be converted to traditional rocking scans, in which for each pixel along the vertical direction (converted to either *q_z_* or *L* in reciprocal units) a column with the intensity for each angle in the selected angular range is exported, as well as appropriate correction factors. The user can then integrate these rocking curves in the traditional manner to obtain structure factors. We have also found this view useful when performing experiments, in that one can easily load a data set and use the sliders to determine a scan range for future scans. Any masks selected in this view are exclusive and pixels inside the masks will be ignored when binning, which is useful for ignoring detector gaps and dead pixels.

### 2.2. Transformed detector view

Even at high energies the image of reciprocal space recorded by the detector is somewhat distorted due to the curvature of the Ewald sphere. The ‘transformed detector view’ in *HAT* corrects for this distortion and assigns reciprocal space coordinates to the image shown in the detector view. The conversion of pixel position to reciprocal space coordinates has been described numerous times (Vlieg, 1997 ▸; Schlepütz *et al.*, 2011 ▸; Smilgies & Blasini, 2007 ▸; Busing & Levy, 1967 ▸), but for completeness we briefly describe the calculations used here, which are somewhat simplified for the horizontal HESXRD geometry.

**Figure 5:**
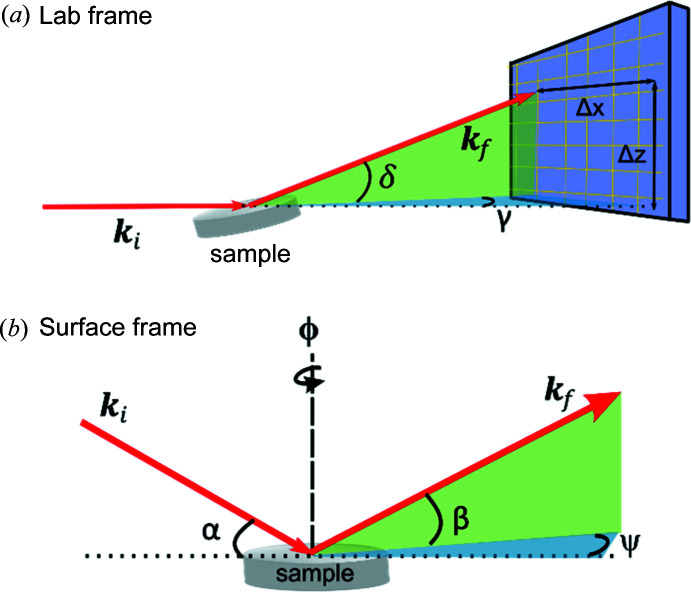
High-energy grazing-incidence horizontal geometry. (*a*) Schematic of the scattering in the laboratory frame, where the incident beam (*k*_i_) lies along the *y* axis and the azimuthal axis ϕ along *z*. (*b*) Schematic of the scattering geometry in the sample frame of reference.

*HAT* assumes that the HESXRD experiment is performed in the grazing-incidence horizontal geometry, shown in Fig. 5 ▸. In the laboratory frame [Fig. 5 ▸(*a*)], we define the angle γ as the azimuthal angle lying along the *x* axis and δ is the altitude along the *z* axis (with *y* then pointing along the direction of the beam). The scattering vector is simply **Q** = **k**_f_ − **k**_i_. In the more natural coordinates of the sample surface [Fig. 5 ▸(*b*)], we can also define the incidence angle of the beam as α and the vertical exit angle as β, the in-plane angle being ψ and the rotation of the sample being ϕ. It is assumed that the sample surface normal has previously been aligned to coincide with the laboratory *z* axis and α is a known angle later applied. In the case of the two reference frames coinciding (when α = 0), the β and ψ angles of each pixel can be calculated via simple trigonometry, as given in equations (1) and (2), where Δ*x* and Δ*z* are the distances along those directions, and *d* is the distance between the sample and the detector:

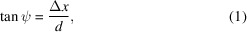




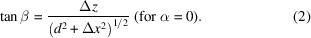

Then for small incidence angles α, as is the case with HESXRD, we can use the small-angle approximation 

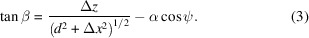

Since the angles for any individual pixel are now given by equations (2) and (3), we can calculate the scattering vector in the surface frame of Fig. 5 ▸(*b*), using the standard angle component form of a vector:

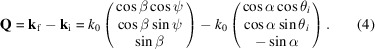

In this case, θ_i_ is the in-plane incidence angle, which is 0, and *k*_0_ = 2π/λ. Now we have the individual components of the scattering vector for each pixel:

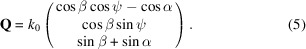

In this scheme, we are invariant to rotation of the sample about the angle ϕ and every pixel has both a *q_y_* component and a small *q_x_* component. A suitable *x* axis is then

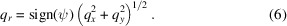

**Figure 6:**
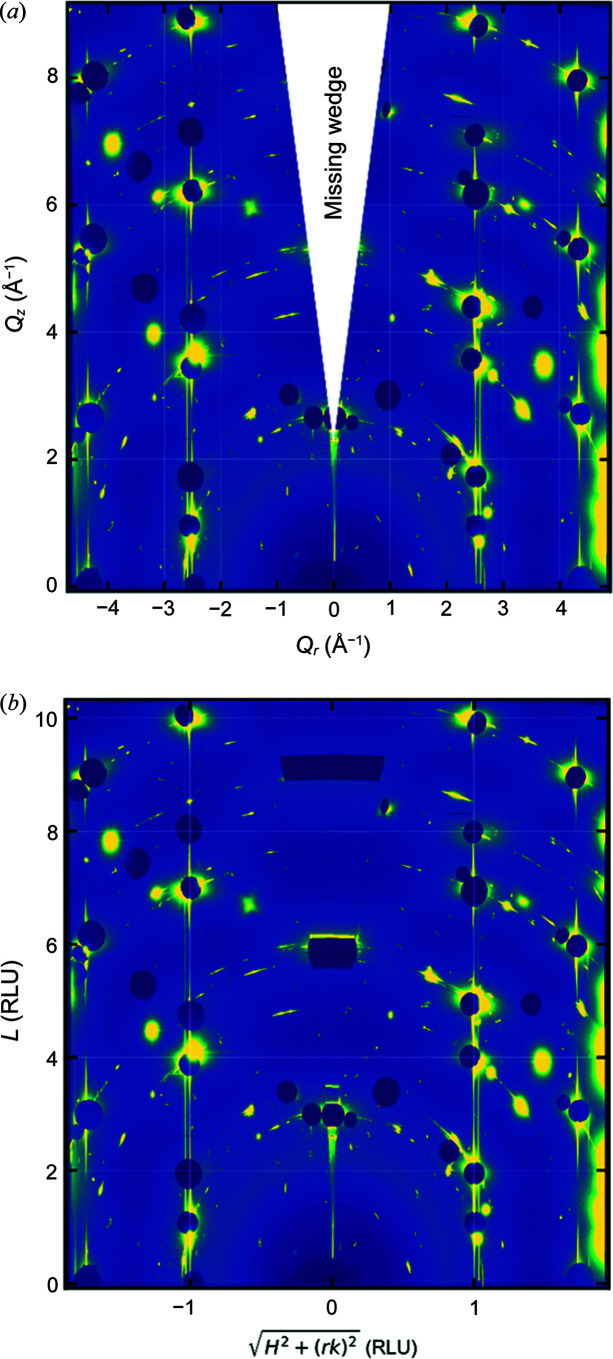
Transformed detector view. (*a*) In units of momentum transfer: in this case the ‘missing wedge’ has been covered by a triangle to clearly highlight it. (*b*) In RLUs: here the intensity where the missing wedge lies is a linear interpolation between the nearest pixels with intensity and can be ignored. The data shown are for an Au(111) electrode in 0.1 *M* NaOH at 0.1 V (versus RHE).

In the ‘transformed detector view’ (when **Q** is selected as the reciprocal unit), *HAT* generates a set of *q_r_* and *q_z_* coordinates for each pixel on the detector. This set of coordinates and the associated intensities (the maximum or average values across the selected angular range) are then interpolated onto a grid. Fig. 6 ▸(*a*) shows such an interpolated image. Note that there is a missing section in the middle of the detector around *q_r_* = 0. This is sometimes referred to as the ‘missing wedge’ and arises from mapping the Ewald sphere to the 2*d* detector plane. Without it the rods would curve at the top of the detector image.

For certain crystal lattice types, it is possible to index such a transformed detector image in reciprocal lattice units (RLUs). This has the advantage that many of the CTRs and Bragg peaks will fall at integer coordinates [see Fig. 6 ▸(*b*)]. The condition for this to make sense in surface coordinates is that the conversion between *q_r_* and *q_z_* is simply the **Q** component divided by the reciprocal lattice vectors. Therefore, *HAT* allows the conversion to RLU when the angles of the two in-plane lattice vectors α_1_ = α_2_ = 90°. Then the vertical axis is *L* = *q_z_*/*b*_3_ and the horizontal axis is

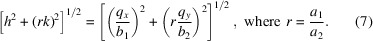

The conventions followed by *HAT* are that the real space lattice vector magnitudes are *a_i_* with angles α_*i*_, and the reciprocal space vectors *b_i_* with angles β_*i*_. These can be entered in the parameter tree [(*k*) in Fig. 3 ▸].

*HAT* can also divide the intensity by several intensity correction factors [equations (8)–(12)] to correct for source polarization [equation (8), we assume the beam is fully horizontally polarized], rod interception [equation (9)], the Lorentz factor since an integration occurs around ϕ [equation (10)], the increased distance of pixels not at the direct beam position due to the curved Ewald sphere on the flat detector [equation (11)] and the non-normal beam inclination at those pixels [equation (12)]. We have previously discussed most of these factors in some detail for the case of a 2 + 3 surface diffractometer with powder diffraction (Abbondanza *et al.*, 2021 ▸), but those that *HAT* uses, presented in equations (8)–(10), are the geometry-independent correction factors given by Smilgies (2002 ▸):

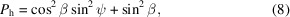




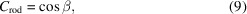




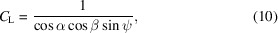




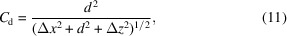




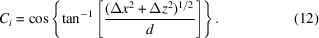

**Figure 7:**
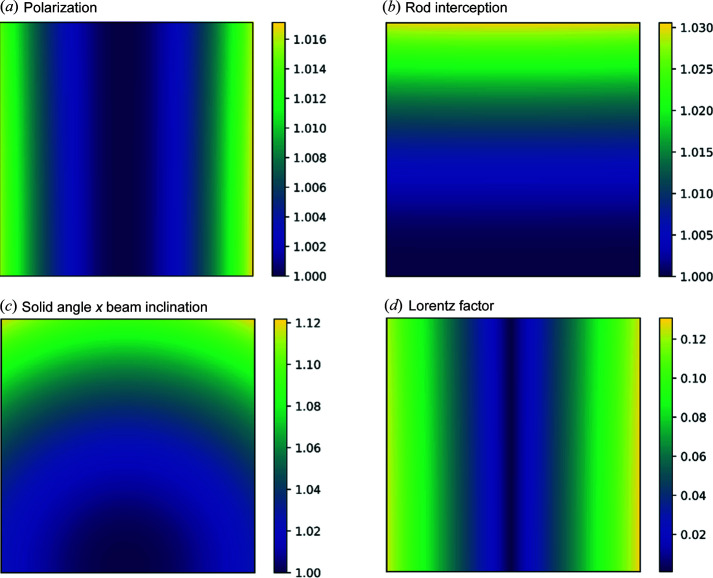
Reciprocal intensity correction factors. Calculated for a 2048 × 2048 pixel (200 × 200 µm) detector at a distance 1.6 m from the sample with an incidence X-ray energy of 73.7 keV which corresponds to the same parameters as the experimental data shown in Figs. 4 ▸ and 6 ▸. The effect of individual correction factors is shown separately and not combined except where indicated.

The general form of these corrections on a detector frame such as Fig. 4 ▸ is shown in Fig. 7 ▸, *i.e.* the intensity which the detector image is multiplied by [the inverse of equations (8)–(12)]. For the transformed detector image, the Lorentz factor is the most significant correction, and strongest in the horizontal direction. In the following section on binning, we integrate in Cartesian coordinates instead of performing an angular integration around ϕ. Therefore this factor is not needed.

### 2.3. 2θ/χ

**Figure 8:**
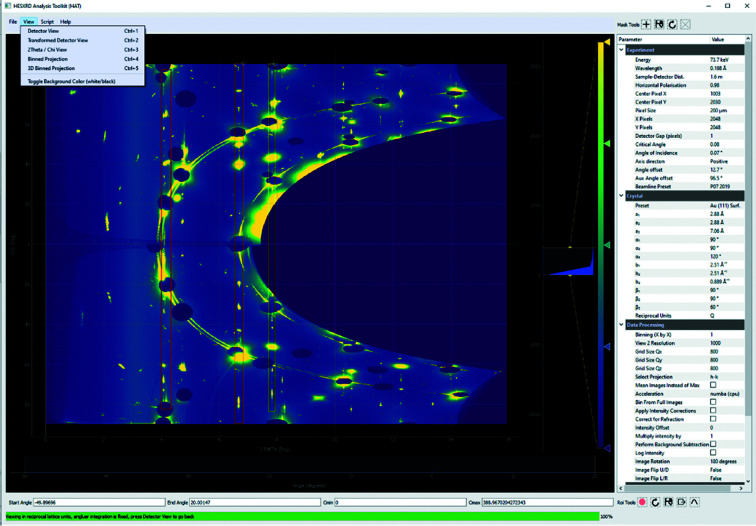
Screenshot of the *HAT* GUI in 2θ/χ mode. This is the same Au(111) data as above, except the data have been transformed such that the angle 2θ forms the horizontal axis and χ forms the vertical axis. In this case the CTRs are now curved and the powder rings are vertical straight lines. Masks can be used to exclude powder rings.

Another common way to transform the detector image is to plot the diffraction angle 2θ along the horizontal axis and the azimuthal angle on the detector plane χ along the vertical axis (Fig. 8 ▸.). As we have previously presented (Abbondanza *et al.*, 2021 ▸), these angles can be simply calculated as

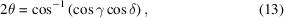




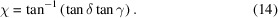

In this view any powder rings will be vertical lines along χ (and the CTRs will be curved). A horizontal box profile can then be placed to produce a traditional 1D powder diffraction pattern (2θ versus summed intensity) over a particular *χ* range. Any masks selected in this mode are exclusive, and it is therefore possible to explicitly remove powder rings from any subsequent binning.

### 2.4. Reciprocal space binning

One of the main features of *HAT* is the ability to perform user-defined reciprocal space binning, which can be used to produce 2D projections from the 3D reciprocal space volume collected by rotating the sample. Consider a *Q_x_* *Q_y_* projection: this is essentially an in-plane map of reciprocal space much like a low-energy electron diffraction (LEED) pattern. *HAT* allows the user to define which pixels to include in the projection via masks, this is useful for avoiding contributions from unwanted features such as powder rings and secondary scattering from beam stops. For an in-plane projection, the masks selected on the transformed detector view indicate the pixels to include and those selected on the detector and 2θ/χ views specify pixels to exclude. For *Q_x_*/*Q_z_*, *Q_y_*/*Q_z_* and 3D projections the pixels to be included are specified as any coordinate that falls both inside a mask on the in-plane view and inside a mask on the transformed detector view (and not inside a detector or 2θ/χ mask).

The *Q_x_*, *Q_y_* and *Q_z_* components for each pixel are calculated as described in the previous section. Then, for each frame (which has an associated ϕ angle) a rotation about the surface normal, ϕ, is applied using equation (15) to give the coordinates (*q*_*x*ϕ_, *q*_*y*ϕ_, *q*_*z*ϕ_) of each pixel in reciprocal space:

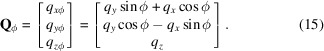

In standard SXRD the orientation of the sample is defined by the U matrix. For *HAT*, we assume the alignment of the sample is reasonably close and the only unknown parameter is the azimuthal orientation of the crystal (ϕ), which the user can specify as an offset. The relationship between **Q**_ϕ_ coordinates and reciprocal lattice coordinates is given as

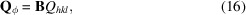

where **B** is the well known matrix that relates the reciprocal lattice to right-handed Cartesian coordinates (17). Internally *HAT* calculates **B** ^−1^ once and then the individual *h*, *k*, *l* components can be quickly computed for each pixel:

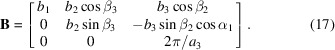

In practice it is simple to determine the required ϕ offset by changing the offset until the rods of an in-plane projection align with the proper integer values of the coordinate axis in the RLU, but one should also check that the Bragg peaks of the rods are at the correct *L* values (depending on the sample symmetry) when there is some ambiguity remaining. For example, the (1 0) rod of the Au(111) surface has Bragg peaks at *L* = 1, 4, 7 (in surface units), whereas the (1 −1) rod has Bragg peaks at *L* = 2, 5, 8; therefore we can determine if the alignment is correct and if it is not all that is required is to offset ϕ by 60°.

To create the 2D projection, an array is created in the computer memory where the number of bins (elements) along each of the coordinate directions is specified by the user. We define the number of bins as *N_x_*, *N_y_* and *N_z_*. The values of the start and end bin are determined from the limits of the masks selected (in units of either *Q*_*x*,*y*,*z*,*r*_ or RLU). For an in-plane map, *x*_min_ = −*x*_max_ since the orientation is unknown. The array indices for a given pixel are then simply

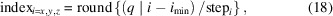




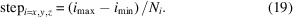

**Figure 9:**
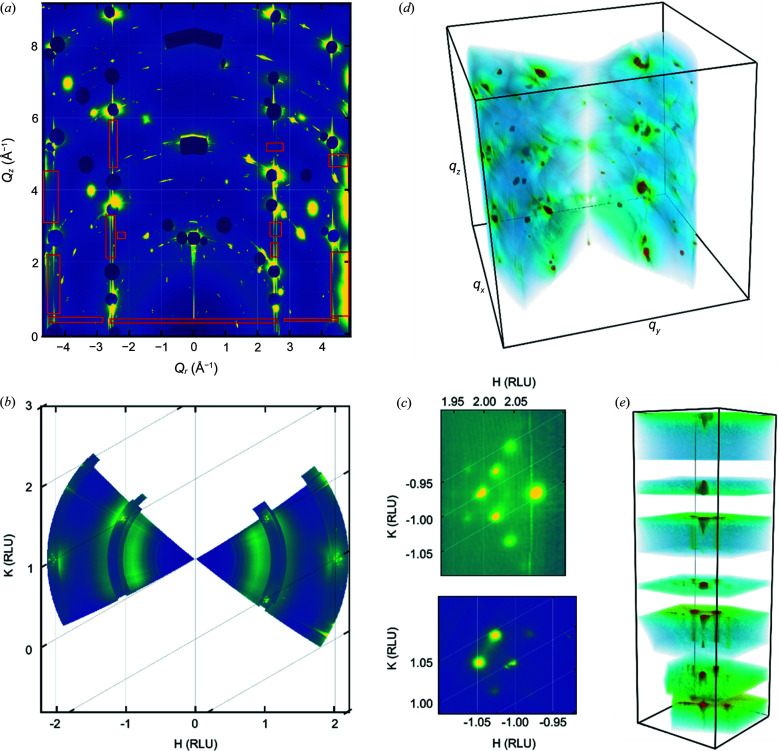
Reciprocal space binning. (*a*) Transformed detector image with several masks (red boxes) indicated in units of momentum transfer. (*b*) In-plane projection generated from the masks in (*a*); this has been plotted in reciprocal lattice units. (*c*) Zoomed-in sections showing the in-plane reciprocal space area around the (2 −1) and (−1 1) CTRs. (*d*) 3D binned representation of the whole reciprocal space volume collected. (*e*) 3D view of the reciprocal space volume around the (1 0) CTR.

For the Au(111) data set shown previously, several masks were selected on the transformed detector view [Fig. 9 ▸(*a*)]. These were used to generate the in-plane map shown in Fig. 9 ▸(*b*). In this map it is clear that the CTRs are close to the integer positions, and they are surrounded by additional spots due to the herringbone surface reconstruction which has previously been shown to be present under these conditions (Gründer *et al.*, 2019 ▸). This is the kind of image one might expect from a high-resolution LEED or spot profile analysis LEED pattern. Fig. 9 ▸(*c*) shows two magnified views around the (2 −1) and (−1 1) CTR positions, where the additional hexagon of spots surrounding the CTR due to the herringbone reconstructions is visible. Each pixel can also be assigned reciprocal space coordinates and binned onto a 3D grid (into voxels). Masks selecting the whole of the transformed detector and in-plane views [Figs. 9 ▸(*a*) and 9 ▸(*b*)] were then selected and used to produce a 3D representation of the reciprocal space volume collected during the 72° rotation [Fig. 9 ▸(*d*)]. A smaller in-plane section was chosen around the (1 0) CTR and used to produce the volume shown in Fig. 9 ▸(*e*). One can see the intensity around of the central CTR and several rods around it due to the herringbone surface reconstruction.

### 2.5. Crystal truncation rod extraction

**Figure 10:**
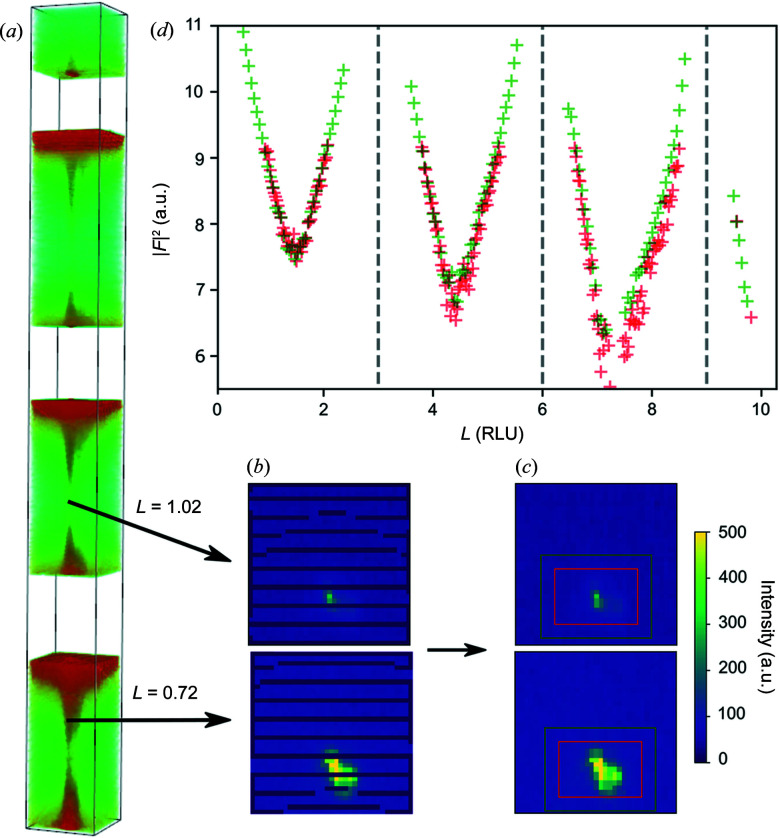
Comparison of (2 −1) CTR extracted from in-plane slices and *hl* projection. (*a*) 3D view of binned pixels around the (2 −1) CTR. (*b*) Slices extracted from CTR at *L* = 0.72 (close to a Bragg peak) and *L* = 1.02 (anti-Bragg position). (*c*) Cubic interpolation is applied to fill in gaps due to the resolution of the measurement. The signal is extracted from the red region of interest and the background from the green region. (*d*) Plot of CTR intensity (structure factor squared) versus *L*. The green CTR markers are extracted by projecting the volume shown in (*a*) on the *hl* plane and drawing a line profile along the CTR and then subtracting the average of line profiles either side. The red markers are extracted from in-plane slices in (*d*).

There are multiple ways one can extract CTRs using *HAT*. The first is to use the profile tool to extract rocking scans and then fit the area under the peaks using another program such as *Ana-Rod*. Secondly, one can select a small angular range over a CTR using the angle range sliders [Fig. 2 ▸(*b*)], change the intensity mode to the mean and then take a profile along the CTR in transformed detector view with intensity corrections enabled. This should be followed by the selection of similarly sized angular ranges either side to measure the background. The third method is to place a mask around the CTR on an in-plane map, which can then be projected onto either a *q_x_* *q_z_* or *q_y_* *q_z_* plane, and then a line profile along the *q_z_* direction can be used to extract the CTR. Background regions can then be selected by moving the mask on the in-plane map to an adjacent region or taking profiles either side of the CTR. This method was used to extract the (2 −1) CTR shown in Fig. 10 ▸(*d*) (green markers). The last method is used to generate a 3D view of the CTR [Fig. 10 ▸(*a*)] which can then be exported as a 3D *NumPy* array. The user can then break the array into slices and extract the intensity by fitting a 2D peak to the CTR signal or using regions of interest [Fig. 10 ▸(*c*)]. Other methods such as peak restoration can also be applied here (Drnec *et al.*, 2014 ▸); for example, we use a cubic interpolation between Figs. 10 ▸(*b*) and 10 ▸(*c*) to fill in gaps. The intensity along the CTR extracted this way is shown in Fig. 10 ▸(*d*) (red markers) and compares well with the previous method. The intensities of the CTRs in Fig. 10 ▸(*d*) dip close to the anti-Bragg positions (between dashed lines). This is due to the interference caused by the surface atoms transitioning between the hexagonally close packed site and the face-centered cubic site.

## 3. Conclusions

Here we have presented *HAT*, a graphical software package that not only allows the rapid assessment of HESXRD data sets but is also a complete reciprocal space binning solution that can be used to create a variety of projections and extract CTRs or other line profiles. *HAT* employs a system of user-selected masks to give precise control over which parts of reciprocal space are included. It is also scriptable and can output publication-quality figures via a *Matplotlib* interface. The software can be accelerated with a GPU and can also run on a laptop computer. We hope that *HAT* will significantly reduce the workload in analyzing HESXRD data.

## 4. Code and data availability

This version (2.0.4) and all future versions can be accessed free of charge under the MIT License from https://github.com/gary-harlow/HESXRD-Analysis-Toolkit. The package is also available to install directly from The Python Package Index (PyPI), typically using the command: $ pip install xrayhat. The example data set (the raw detector images and metadata files) used in this manuscript is freely available for download from the figshare repository (Harlow, 2022 ▸). The latest documentation on the project can also be found at https://xray-hat.readthedocs.io/en/latest/.

## Figures and Tables

**Table 1:** Comparison of the advantages and disadvantages of HESXRD

Advantages of HESXRD	Disadvantages of HESXRD
Simpler measurement geometry. Typically, only the sample azimuthal angle needs to be changed after alignment.	Need to place beam stops; therefore the signal close to the Bragg peak can be difficult to measure.
Distortion-free regions of reciprocal space. The CTRs look directly at the detector, which makes initial assessments of the data much easier and facilitates our understanding of the technique.	Reduced scattering cross section. All elements have a smaller elastic cross section at higher energies which decreases with the wavelength squared.
Fast measurement of many CTRs up to high **Q**, as one can perform a quick azimuthal fly scan.	Increased data processing overhead. This software attempts to resolve this, but regardless there is certainly a vast increase in data storage required.
Can penetrate more difficult sample environments due to the high energies and low exit angles.	Incidence angle still needs to be changed to measure the specular CTR.
Faceting or rods from faceted nanoparticles are easier to identify.	
